# Cognitive control training enhances the integration of intrinsic functional networks in adolescents

**DOI:** 10.3389/fnhum.2022.859358

**Published:** 2022-11-24

**Authors:** Raihyung Lee, Seyul Kwak, Dasom Lee, Jeanyung Chey

**Affiliations:** ^1^Department of Psychology, Seoul National University, Seoul, South Korea; ^2^Department of Psychology, University of California, Los Angeles, Los Angeles, CA, United States; ^3^Department of Psychology, Pusan National University, Busan, South Korea

**Keywords:** cognitive control, cognitive training, adolescence, development, resting-state fMRI, graph theory

## Abstract

**Introduction:**

We have demonstrated that intensive cognitive training can produce sustained improvements in cognitive performance in adolescents. Few studies, however, have investigated the neural basis of these training effects, leaving the underlying mechanism of cognitive plasticity during this period unexplained.

**Methods:**

In this study, we trained 51 typically developing adolescents on cognitive control tasks and examined how their intrinsic brain networks changed by applying graph theoretical analysis. We hypothesized that the training would accelerate the process of network integration, which is a key feature of network development throughout adolescence.

**Results:**

We found that the cognitive control training enhanced the integration of functional networks, particularly the cross-network integration of the cingulo-opercular network. Moreover, the analysis of additional data from older adolescents revealed that the cingulo-opercular network was more integrated with other networks in older adolescents than in young adolescents.

**Discussion:**

These findings are consistent with the hypothesis that cognitive control training may speed up network development, such that brain networks exhibit more mature patterns after training.

## Introduction

Cognitive control, the ability to guide behavior in a goal-directed fashion, is a key requirement for everyday tasks, which improves throughout adolescence (Huizinga et al., 2006; Luna et al., 2010). Given that cognitive control is a strong predictor of crucial life outcomes such as academic achievement and mental health (Paus et al., 2008; Moffitt et al., 2011), numerous studies have aimed at improving cognitive control through cognitive training interventions in adolescents (for review, see Karbach and Unger, 2014). Despite growing interest in cognitive training and reports of its effectiveness, relatively little is known about the underlying mechanisms of these training effects. This is because of the difficulty in combining longitudinal neuroimaging analysis with controlled behavioral interventions, especially in subjects such as children and adolescents (Rueda et al., 2012; Astle et al., 2015). The goal of the present study was to examine whether and how cognitive control training alters brain networks during adolescence.

Cognitive control requires the coordination of executive components such as task-set switching, adaptive gating, working memory, and response inhibition, and thus, it involves widely distributed brain circuitries (Lenartowicz et al., 2010; Cole et al., 2013). Similarly, researchers have found that adolescent cognitive control is not attributable to the isolated operations of single brain regions but rather that it is dependent on an interplay between large-scale brain networks (Dwyer et al., 2014; Luna et al., 2015). Brain networks associated with cognitive control include the fronto-parietal network (FPN) and cingulo-opercular network (CON), which are deemed task-control networks; the cerebellar network (CBN), which provides error-related feedback to task-control networks; and the default mode network (DMN), which is a task-negative network (Dosenbach et al., 2008; Fair et al., 2009). Cognitive control is supported by the ability for these specialized functional networks to collaborate and flexibly integrate information (Cole and Schneider, 2007). This fact suggests that the training of higher-order cognitive skills, such as cognitive control, is likely to have a broader impact on the brain at a systematic network level, which cannot be fully captured by inspecting single brain regions or tracts (Taya et al., 2015; Caeyenberghs et al., 2016).

Existing studies have demonstrated changes in brain functional networks among adults after various types of cognitive training, such as working memory training (Takeuchi et al., 2013), reasoning training (Mackey et al., 2013) and mnemonic training (Dresler et al., 2017). It should be noted, however, that the same training could have different outcomes in adolescents depending on the stage of brain development in which the change takes place. While training in adults largely modifies the existing neural architecture, training in adolescents may still influence the ongoing construction of neural structures (Galván, 2010). Thus, training effects in adolescents are best understood in the context of the developing brain because they result from an interaction between learning and brain maturation (Jolles and Crone, 2012). One possible mechanism of how training affects the developing brain is that it may accelerate the maturational process, such that brain structure and function become more similar to those of adults after training (Jolles and Crone, 2012). In one study, for example, young adolescents exhibited a more mature pattern of fronto-parietal brain activation after intensive working memory practices (Jolles et al., 2012). A similar effect was observed in children who showed a more adult-like scalp distribution of event-related potentials after participating in an executive attention training (Rueda et al., 2005). Because few studies have examined the impact of training on the brain in children or adolescents, more work is needed to explain the process by which cognitive training rewires the developing brain.

Developmental changes in brain functional networks have been investigated using a powerful graph theoretical approach applied to intrinsic connectivity at rest (Vértes and Bullmore, 2015). The maturation of functional networks during adolescence is primarily characterized by refinements of network structures that are already present in infancy and childhood (Grayson and Fair, 2017). Multiple reports have found that basic network topologies are evident early in development, but continue to evolve during adolescence in ways that support the emergence of more complex cognitive abilities (Cao et al., 2014; Gu et al., 2015). One core principle of network maturation, which is particularly critical to the development of cognitive control, is greater integration among networks (Luna et al., 2015). Children display specialized networks with similar organization structure to those of adults (Power et al., 2012; Fair et al., 2013), but the integration between those networks continues to strengthen during adolescence (Hwang et al., 2013). A recent study directly investigating the neural basis of cognitive control development has also found that the foundational organization of brain functional networks does not change from children to adults (Marek et al., 2015). However, the process of network integration, particularly the integration of the CON with other brain networks, has been shown to continue throughout adolescence. Importantly, this increased cross-network integration of the CON underlies behavioral improvements in cognitive control (Marek et al., 2015).

Here, we trained typically developing adolescents on a set of cognitive control tasks, and examined how their resting-state functional networks changed by applying graph theoretical analysis. Specifically, we hypothesized that cognitive control training would accelerate the typical brain network development of adolescents, and thereby promote the integration of functional networks. This is consistent with previous studies showing that cognitive training leads to neurophysiological changes that seemingly expedite brain maturation (Rueda et al., 2005; Jolles et al., 2012). Fifty-one young adolescents (age, 13.19 ± 0.65 years) were randomly assigned to the training or the control condition, and they underwent resting-state functional magnetic resonance imaging (rs-fMRI) before and after training. We analyzed the *degree* of brain functional networks, which is a commonly used graph theoretical measure, and examined how the degree of brain functional networks changed as a result of training. We used the ADHD-200 dataset, comprising rs-fMRI scans from 58 typically developing older adolescents (age, 17.17 ± 1.22 years), as a contrast group. The network degree of these older adolescents was compared to that of our young adolescents to test the hypothesis. Finally, we examined how changes in brain networks after training relate to gains in behavioral performance measured outside the scanner.

## Materials and methods

### Participants

Sixty-four healthy participants aged 12–14 years enrolled in the study. Participants were recruited through online posting to community schools and private academies located in the Seoul metropolitan region. A phone screen was used to assess their medical history at the time of recruitment. Participants previously diagnosed with a neurological or psychiatric illness were excluded. All participants were right-handed, free from prior head injury, and eligible for the magnetic resonance imaging (MRI) environment. Before participation, all subjects provided informed written consent approved by the institutional review board of the Seoul National University.

Participants underwent MRI and cognitive tests before and after 6 weeks of training. Three participants who dropped out of training and eight participants who failed to complete 80% of the required training schedule were excluded from the analysis. Two additional participants were excluded due to acquisition problems (i.e., scanner malfunction and poor visual acuity). We found no subject whose head motion exceeded 3 mm translation or 3 degrees rotation. Final data are reported for 25 participants (11 females) in the training group and 26 participants (11 females) in the control group (age, 13.19 ± 0.65 years).

Data regarding older adolescents were provided by the ADHD-200 Consortium (Brown et al., 2012), who maintain an open access dataset comprising data from children with and without ADHD, aggregated from eight different sites. The current study used data from the University of Pittsburgh and the New York University (NYU) Child Study Center, the sites that had the largest number of late adolescents without ADHD. Detailed descriptions of the participant recruitment procedures and selection criteria are available online at http://fcon_1000.projects.nitrc.org/indi/adhd200/.

The original Pittsburgh and NYU datasets consisted of resting-state data collected from 361 subjects: 98 from Pittsburgh and 263 from NYU. We restricted our analysis to typically developing adolescents whose ages ranged from 15 to 18 years (*n* = 86). Of those, four subjects who had fewer resting-state scans than other subjects were excluded. We also excluded two left-handed subjects and two subjects with excessive head motion (>3 mm translation or >3° rotation). All subjects had IQ scores within the normal range. According to the protocol shared online, ADHD-200 participants did not engage in any type of cognitive intervention throughout the course of their participation. The final set of subjects consisted of 58 individuals (31 females; age, 17.17 ± 1.22 years). Subject exclusion criteria for the ADHD-200 dataset are summarized in Table 1.

**TABLE 1:** Subject exclusion criteria for ADHD-200 dataset.

Exclusion criteria	No. of subjects excluded	
Original dataset	University of Pittsburgh (*n* = 98)	NYU Child Study Center (*n* = 263)
Age < 15 or > 18 years	53	222
ADHD diagnosed	3	17
Fewer fMRI scans	1	3
Left-handedness	1	1
Excessive motion	1	1
Subjects included	39 (21 females)	19 (10 females)

### Cognitive assessment

A battery of cognitive tests were administered pre- and post-training. The tests were selected that tap into different aspects of cognitive control. It included the following: (A) The Stroop task measures response inhibition and interference resolution (Shin and Park, 2007). The outcome variables are the number of items completed in 45 s for each condition (W, word score; C, color score; CW, color–word score). The interference was quantified, as Golden (Golden, 1978) proposed, by first calculating a predicted color–word score (PCW) on the basis of the word (W) and color (C) scores: PCW = 45/{[(45 × W) + (45 × C)]/(W × C)} = (W × C)/(W + C). This score was then subtracted from the actual color–word score (CW) to calculate the interference score (I = CW – PCW). Because a higher actual color–word score relative to the predicted score suggests one’s performance was better than anticipated, the higher interference control score indicates better interference control; (B) the Trail Making Test (TMT) comprised parts A and B. In part A, the subjects used a pencil to connect a series of 25 encircled numbers in numerical order. In part B, the subjects connected 25 encircled numbers and letters in numerical and alphabetical order, alternating between the numbers and letters. TMT reflects set-switching and executive control, and the interference score was calculated as follows: Interference score = (TMT-B score – TMT-A score) / TMT-A score (Lee et al., 2002). Because TMT-B captures the additional cost for alternating switches, the higher interference score indicates poorer interference control (Sánchez-Cubillo et al., 2009); (C) The Digit Span test was measured for forward- and reverse-order recall of digit sequences. Digit sequences were presented beginning with a length of 2 digits, and two trials were presented at each increasing list length. Testing ceased when the participant failed to accurately report either trial at one sequence length or when the maximal list length was reached. The total number of lists reported correctly was combined across forward span and backward span to calculate the test score. The Digit Span test measures verbal working memory (Hwang et al., 2012); (D) In the Symbol Span test, subjects were shown an increasing number of simple visual designs. After the display was removed, the subjects were asked to identify the correct designs while also stating their correct presentation order from left to right. The total number of designs reported correctly gave the test score. The Symbol Span test measures visual working memory (Chey et al., 2012); (E) In the Arithmetic test, subjects were verbally presented with hypothetical scenarios involving simple arithmetic calculations and were asked to calculate the correct answers. The number of questions that subjects correctly answered produced the test score. The Arithmetic test assesses working memory and mathematical reasoning (Kwak et al., 2011); (F) In the Block Design test, the subjects were asked to rearrange the three-dimensional blocks that have various color patterns on different sides to match a pattern that they were presented with. The items in the Block Design test were scored both by accuracy in matching the pattern and by speed in completing each item (Kwak et al., 2011); (G) In the Matrix Reasoning test, the subjects were shown colored matrices of visual patterns with something missing and were asked to select the missing piece from a range of options. The total number of questions answered correctly gave the test score. The Block Design test and the Matrix Reasoning test evaluate perceptual organization and perceptual reasoning, both considered adequate measures of fluid intelligence (Kwak et al., 2011).

Three subjects did not complete the TMT because they did not know the order of the Korean alphabet. Also, STROOP score for one subject was missing because the subject had a mild color weakness. Missing values were imputed with the aregImpute function in the Hmisc package for R. Missing values were predicted by other cognitive test scores using bootstrap and predictive mean matching (For the details of the imputation method, see Harrell and Harrell, 2018).

Additionally, we collected the Perceived Stress Scale (Cohen et al., 1983) scores during the pre- and the post-training visits, since stress has been associated with changes in FP connectivity (Liston et al., 2009). Further, because juvenile impulsivity has also been reported to alter the resting functional connectivity (FC) (Shannon et al., 2011), we collected the UPPS-P Impulsive Behavior Scale (Whiteside and Lynam, 2001) scores at both time points.

### Cognitive control training

We modified a multicomponent cognitive control training program (Kim et al., 2017) for the adolescents. The training program consisted of seven computerized tasks that could be performed via the internet. Given that cognitive control comprises a set of cognitive components including task-set switching, updating, working memory, and response inhibition (Miyake et al., 2000), each task was designed to tap into one or more of these components. The procedure of each task and cognitive components they are designed to target are summarized in Table 2.

**TABLE 2:** A description of the tasks for training cognitive control.

Task	Description	Related components
Location-Number Stroop	Participants were presented with number matrices (4 × 4 and 5 × 5). They were asked to report the horizontal location of the number not the number itself. Every trial in this task was incongruent (e.g., ‘4’ in second location).	Inhibition
Rock-Paper-Scissor	Participants were presented with one of the three hand shapes constituting Rock-Paper-Scissor, and they were instructed to win or lose the game depending on the color of the hand. There were four conditions; participants must win to the pink hand, lose to the blue hand, apply the rule same as that of the previous trial to the yellow hand, apply the rule opposite to that of the previous trial to the green hand.	Inhibition, shifting, memory updating
Counting clovers	On the right and left sides of the screen two groups of clovers (green group and orange group) were presented simultaneously. The color of the clover group on each side was randomly changed (i.e., sometimes green on the left side and orange on the right side sometimes vice versa). The task was to respond which group has a greater number than the other (right button for green group and left button for orange group irrespective of their location). At the same time participants had to press the space bar whenever the total sum of the numbers was 3, 5, 7, 9, and 11.	Inhibition, dual-tasking
Updating	There were three versions of the updating task (i.e., ‘color,’ ‘location,’ and ‘letter’). In the color updating task, colored circles were presented serially and randomly with varied length. The task was to recall the last several colors as quickly as possible. Locations and Korean letters instead of colored circles were used in the other two versions.	Inhibition, memory updating
Star and Moon	There were four types of stimuli comprising the combinations of two colors (yellow and blue) and two shapes (star and moon). Participants were instructed to respond to stimuli only based on colors or shapes at the beginning of each set of trials.	Inhibition, shifting

Both groups performed the training at home. The training lasted 6 weeks, with the training group performing five sessions a week (30 sessions in total) and the control group two sessions a week (10 sessions in total). For the training group, each session comprised three different tasks with a total duration of approximately 30 min. The tasks were selected for each session in a way all seven tasks were evenly distributed across sessions. The task difficulty was adjusted on a trial-by-trial basis according to the individual’s improvement as the sessions proceeded. We used the active control group to control for placebo effect (Klingberg, 2010). For the control group, each session comprised two tasks, but took less than 10 min to complete because the tasks were designed not to progress to more difficult levels despite improvement. In other words, the control group performed the same set of tasks as the training group, but the task difficulty was fixed at the lowest level. Each individual’s training record was monitored online by the researchers. If an individual showed signs of the lack of engagement with the tasks (e.g., too long response time for each trial, too many incorrect responses in a row), the researchers contacted the trainee and ensured that they grasp rules of the tasks correctly and pay close attention while performing the tasks. Eight participants who failed to complete 80% of the required training schedule were excluded from the analysis.

The duration and intensity of the present training was determined on the basis of the existing cognitive training literature. Studies show variations in the duration and intensity of training regimes, which understandably impact the success of training interventions (Jaeggi et al., 2008; Alloway et al., 2013). Most published training regimes comprise approximately 20 training sessions each lasting approximately 30 min; therefore, our training duration should be sufficient to induce behavioral changes (von Bastian and Oberauer, 2013; Noack et al., 2014). With respect to the intensity of training, task difficulty was designed to significantly tax the cognitive resources of young adolescents. By utilizing adaptive training algorithms, we kept the task challenging throughout the training phase, and thereby maximized the engagement of cognitive control in trained subjects.

### Image acquisition and pre-processing

Anatomical and functional MRI scanning was performed on a 3T Siemens Tim Trio at the Brain Imaging Center at the Seoul National University. Subjects’ heads were fixed using foam padding and a 32-channel head coil. T1-weighted structural images were collected using a magnetization-prepared rapid gradient echo (MPRAGE) sequence (TR = 2300 ms, TE = 2.36 ms, 1.0 × 1.0 × 1.0 mm voxels, FOV = 256 mm). During the 6-min resting-state scan, the participants were asked to remain relaxed with their eyes open while gradient echo EPI images were acquired (TR = 2200 ms, TE = 30 ms, 33 axial slices, 3.0 × 3.0 × 3.5 mm voxels, flip angle = 79°, FOV = 240 mm, GRAPPA factor 2). Prior to the resting-state scan, the subjects underwent two sessions of a cognitive control task fMRI. Data from the task fMRI sessions will be reported elsewhere.

Image pre-processing was performed using the Statistical Parametric Mapping toolbox (SPM12^1^) running under Matlab R2017a (Mathworks). Each subject’s EPI images were unwarped using field maps and were realigned using a six-parameter (rigid body) spatial transformation with the first image as a reference. The images were then corrected for differences in slice timing acquisition. Spatial normalization was achieved via the Diffeomorphic Anatomical Registration through Exponentiated Lie Algebra procedure (Ashburner, 2007). EPI images were co-registered to T1 images and a sample-specific template was created using all subjects’ T1 images. Then, the deformation of the T1 images to a sample-specific template was calculated and applied to normalize the EPI to standard space. The images were finally smoothed with an 8-mm full-width at half-maximum Gaussian kernel.

The Pittsburgh and NYU data were acquired using an imaging protocol similar to that used for the collection of the Seoul National University data. For the Pittsburgh data, MRI scanning was performed on a 3T Siemens Tim Trio at the University of Pittsburgh Medical Center Magnetic Resonance Research Center. T1-weighted images were collected using a MPRAGE sequence (TR = 2100 ms, TE = 3.43 ms, 1.0 × 1.0 × 1.0 mm voxels). During the 5-min resting-state scan, gradient-echo EPI images were acquired with the following parameters: TR = 1500 ms, TE = 29 ms, 29 axial slices, 3.1 × 3.1 × 4.0 mm voxels, GRAPPA factor 2. For the NYU data, imaging was performed on a 3T Siemens Allegra at the NYU Center for Brain Imaging. T1-weighted images were acquired using a MPRAGE sequence (TR = 2530 ms, TE = 3.25 ms, 1.3 × 1.0 × 1.3 mm voxels). A 12-min resting-state scan comprising two sessions was acquired using a multi-echo EPI sequence (TR = 2000 ms, TE = 15 ms, 33 axial slices, 3.0 × 3.0 × 4.0 mm voxels). More detailed descriptions of the ADHD-200 imaging protocol can be found in the literature (Bellec et al., 2017).

We applied standard pre-processing procedures to the ADHD-200 fMRI data using SPM12 as follows: the images were slice-time and motion corrected, registered to MNI space using non-linear transformation, and smoothed with an 8-mm Gaussian kernel.

### Network definition

**FIGURE 1:**
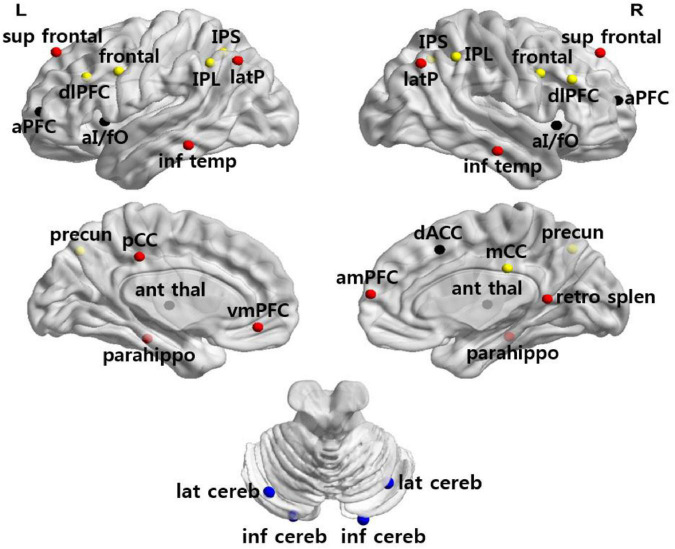
Anatomical location of ROI. Regions are colored by network membership (yellow, FPN; black, CON; red, DMN; blue, CBN) and overlaid on a cortical surface.

We used 34 previously defined regions of interest (ROI) comprising four functional networks (i.e., FPN, CON, DMN, CBN) whose coordinates were derived from FC mapping and meta-analytic techniques (Dosenbach et al., 2008; Fair et al., 2009; Figure 1). These networks were chosen for their central involvement in exerting cognitive control. ROI were generated as 7.5-mm radius spheres around a center coordinate. Each ROI represented a node of the network. The MNI coordinates and the corresponding regions for all sets of ROI are listed in Table 3.

**TABLE 3:** Regions of interest, their MNI coordinates, and functional properties.

Regions of interest (ROI)	Abbreviations	Coordinates			Functional network	Network color
		*x*	*y*	*Z*		
Dorsolateral prefrontal cortex	dlPFC	–45.04	28.22	31.49	Fronto-Parietal	Yellow
Dorsolateral prefrontal cortex	dlPFC	47.88	28.55	29.87	Fronto-Parietal	Yellow
Frontal	frontal	–42.77	8.23	35.67	Fronto-Parietal	Yellow
Frontal	frontal	45.82	8.54	34.13	Fronto-Parietal	Yellow
Mid cingulate cortex	mCC	1.57	–26.27	31.60	Fronto-Parietal	Yellow
Inferior parietal lobule	IPL	–53.35	–49.24	41.54	Fronto-Parietal	Yellow
Inferior parietal lobule	IPL	56.91	–43.97	45.86	Fronto-Parietal	Yellow
Intraparietal sulcus	IPS	–31.63	–57.05	48.66	Fronto-Parietal	Yellow
Intraparietal sulcus	IPS	34.24	–59.26	44.40	Fronto-Parietal	Yellow
Precuneus	precun	–7.88	–71.31	44.07	Fronto-Parietal	Yellow
Precuneus	precun	12.67	–67.84	45.61	Fronto-Parietal	Yellow
Anterior prefrontal cortex	aPFC	–29.21	57.15	7.07	Cingulo-Opercular	Black
Anterior prefrontal cortex	aPFC	30.32	57.13	15.02	Cingulo-Opercular	Black
Anterior insula/frontal operculum	aI/fO	–36.76	16.73	–0.01	Cingulo-Opercular	Black
Anterior insula/frontal operculum	aI/fO	39.92	19.03	–2.67	Cingulo-Opercular	Black
Dorsal anterior cingulate	dACC	0.56	16.87	45.28	Cingulo-Opercular	Black
Anterior thalamus	ant thal	–11.77	–13.81	4.83	Cingulo-Opercular	Black
Anterior thalamus	ant thal	12.01	–13.63	5.52	Cingulo-Opercular	Black
Anteriormedial prefrontal cortex	amPFC	2.19	61.08	12.87	Default	Red
Ventromedial prefrontal cortex	vmPFC	–2.39	42.72	–11.01	Default	Red
Superior frontal cortex	sup frontal	–13.52	47.22	49.24	Default	Red
Superior frontal cortex	sup frontal	19.98	46.28	48.76	Default	Red
Inferior temporal	inf temp	–64.94	–35.43	–16.78	Default	Red
Inferior temporal	inf temp	71.12	–17.93	–20.83	Default	Red
Parahippocampal	parahippo	–22.84	–27.94	–19.36	Default	Red
Parahippocampal	parahippo	27.96	–27.55	–18.02	Default	Red
Posterior consulate cortex	pCC	–0.46	–33.00	40.15	Default	Red
Lateral parietal	latP	–48.96	–66.24	43.14	Default	Red
Lateral parietal	latP	59.07	–65.86	41.27	Default	Red
Retro splenial	retro splen	4.60	–51.94	9.44	Default	Red
Lateral cerebellum	lat cereb	–33.67	–71.87	–29.40	Cerebellar	Blue
Lateral cerebellum	lat cereb	34.38	–66.31	–31.11	Cerebellar	Blue
Inferior cerebellum	inf cereb	–19.63	–85.00	–32.82	Cerebellar	Blue
Inferior cerebellum	inf cereb	20.35	–86.99	–33.30	Cerebellar	Blue

Functional connectivity analysis was performed using the CONN toolbox v17 (Whitfield-Gabrieli and Nieto-Castanon, 2012), implemented with SPM12. For both training and ADHD-200 datasets, an exact noise reduction method called CompCor was used to extract the principal components from white matter and cerebrospinal fluid signals (Behzadi et al., 2007), which were entered as confound regressors in a subject-level GLM. This approach corrects for physiological and other spurious noise without relying on global signal regression, which has been shown to introduce artifactual anticorrelations (Murphy et al., 2009; Chai et al., 2012). Given the importance of reducing motion artifacts, we used the Artifact Rejection Toolbox (ART^2^) to detect outlier image frames based on brain activation and head movement (motion scrubbing; Power et al., 2014). In order to detect outlier frames in brain activation data, the global signal was calculated across time and *Z*-normalized. Outliers were defined as frames in which the global signal fell outside 5 SD. Similarly, motion outliers were defined as points in which the frame-wise measures of motion exceeded 0.9 mm. In addition to the six realignment parameters (three translation and three rotation parameters) and their first order derivatives, outlier frames identified using ART were included as confound regressors. The data underwent linear detrending and a temporal filter of 0.009 and 0.08 Hz was applied to focus on low frequency fluctuations (Fox et al., 2005). For each participant, a time series was calculated by averaging the BOLD signal across all voxels within each ROI. Pearson correlation coefficients were computed for each pair of ROIs, and were Fisher-transformed to produce normally distributed values. The resulting 34 × 34 symmetric correlation matrices represent the networks of nodes and edges of each participant’s intrinsic FC profile. Graph theoretical-based analyses were performed on these networks. All statistical analyses were uncorrected for multiple comparisons.

### Graph theoretical analysis

Utilizing graph theoretical analysis, we tested the hypothesis that cognitive control training would facilitate the integration of functional networks in normal adolescents. To define network integration, we used a basic and important graph measure, *degree.* The degree of an individual node is equal to the number of links connected to that node. The neurobiological interpretation of degree is straightforward; brain regions of high degree are interacting, structurally or functionally, with many other regions (Rubinov and Sporns, 2010). Brain regions in networks may either contain links to regions within the same network (within-network links) or contain links to regions belonging to other networks (between-network links). A network whose nodes have many links to other networks—that is, one containing nodes with a high between-network degree—facilitates interactions among different networks. Thus, in the context of network integration, a network with a high between-network degree is interpreted as being highly integrated.

To investigate changes in network integration after training, we calculated the mean degree for the FP, CO, CB, and DM networks for each subject. We took the absolute value of all negative weights and thresholded each subject’s connectivity matrices by network density, ranging from the strongest 15–60% of pairwise connections. Connections above the threshold were binarized and the average number of links connected to nodes in each network was calculated. Group × Time ANOVA was then conducted to examine whether the increases in the mean degree of networks were greater in the training group than in the control group.

To calculate the degree for each of the four networks as described above, the nodes were grouped by the network to which they were assigned in the predefined ROI partition (Table 3). However, it is possible that the network organization of our subjects does not follow the predefined ROI partition scheme. To ensure that our subjects’ networks are organized in the same way as the ROI sets (partitioned into four networks), we performed a modularity analysis. A modularity analysis decomposes the whole-brain network into several distinct modules. Modules are characterized by nodes that work more closely together than with nodes belonging to other modules (Newman and Girvan, 2004). When applied to brain FC matrices, modules correspond to collections of strongly interconnected brain regions sub-serving common functions and, therefore, are regarded as functional brain networks (Meunier et al., 2009; Stevens et al., 2012). To perform a modularity analysis, we first averaged all 51 subjects’ 34 × 34 connectivity matrices. We then partitioned average connectivity matrices into modules using a popular greedy modularity-maximization algorithm known as the Louvain algorithm (Blondel et al., 2008) across network densities. The Louvain algorithm has been verified to be one of the most accurate module detection algorithms (Lancichinetti and Fortunato, 2009). The resulting modular structure represents the functional network organization of our subjects. The similarity between the network organization of our subjects and the predefined ROI partition was then evaluated.

To interpret the meaning of the changes in network degree following training, we calculated the mean degree of each network in older adolescents from the ADHD-200 dataset. We conducted independent *t*-tests to examine whether there was any difference in network degree between older and our younger adolescents before training.

Finally, to examine brain–behavior correlations, we tested whether the increases in the mean degree of networks were correlated with improvements in cognitive test performance. We performed Pearson correlation analysis to investigate whether the improvements in cognitive test performance were associated with significant increases in the mean degree of networks in the abovementioned Group × Time ANOVAs.

As a Supplementary Analysis, in addition to network degree, we explored another graph theoretical construct, participation coefficient (PC), to examine the effects of training on network integration. PC is a graph theoretical construct that quantifies the level to which a node establishes between-network links compared with within-network links; nodes with many distributed between-network links would have higher PC whereas nodes whose links are mostly restricted to their own networks would have lower PC (Guimerà and Nunes Amaral, 2005; For a more detailed and technical description, see Supplementary Analysis). A network containing nodes with high PC values is likely to promote the integration of networks. We calculated the mean PC for each of the four networks and conducted Group × Time ANOVA to examine whether the increases in the mean PC of networks were greater in the training group than in the control group.

## Results

### Demographics and behavioral measures

The training group and the control group were well matched for age, sex, scores on cognitive tests before training, and number of days between tests. The subjects were within a tight age range, which reduced the effects of maturation on cognitive control and resting-state connectivity patterns. Both groups were also matched on stress and impulsivity levels, and neither group displayed a change in either of these variables before and after training (Table 4). Given that head motion confounds analyses of resting-state connectivity (Power et al., 2012; van Dijk et al., 2012), we confirmed that neither mean relative head displacement nor number of frames with a displacement of >0.5 mm changed between pre-and post-training for either group (control, *p* > 0.4; training, *p* > 0.3) or differed between groups at either time point (pre-training, *p* > 0.3; post-training, *p* > 0.3; Mackey et al., 2013).

**TABLE 4:** Participant information.

	Training group (*n* = 25)	Control group (*n* = 26)	t/χ^2^	*p*
	Mean (*SD*)	Mean (*SD*)		
Age	13.22 (0.74)	13.15 (0.58)	–0.33	0.741
Sex (Female)	11	11	<0.01	1.000
Test–retest interval (Days)	64.5 (9.81)	65.92 (10.03)	–0.51	0.612
Perceived stress				
Time 1	20.23 (6.87)	19 (5.34)	0.72	0.478
Time 2	19.5 (7.66)	18.28 (5.83)	C^‡^ 0.6 T^‡^ 0.71	0.556 0.484
Impulsivity				
Time 1	136.42 (16.26)	132.24 (18.13)	0.87	0.391
Time 2	138.04 (18.66)	127.72 (21.76)	C^‡^ –0.63 T^‡^ 1.72	0.533 0.100

^‡^Paired *t*-test was used to assess the changes between Time 1 and Time 2 in control and training groups.

To ensure the quality of the training protocol employed in the current study, we also investigated whether there were improvements in performance on training tasks. The training performance was measured by the level and accuracy of the administered tasks and these data were fitted to a linear mixed effects model. The accuracy and maximum levels obtained by each participant in the training group during each training session were expected to show improvements if the training procedure was properly applied. After the completion of the training, the level of Location–Number Stroop and Counting Clovers upgraded significantly [*t*(24) = 2.72, *p* = 0.013, *r* = 0.48; *t*(24) = 3.89, *p* = 0.004, *r* = 0.62], and the level of Updating (location) showed tendency to increase [*t*(24) = 1.88, *p* = 0.089, *r* = 0.36]. In addition, the accuracy of Updating (letter) and Counting Clovers significantly improved [*t*(24) = 4.40, *p* < 0.001, *r* = 0.67; *t*(24) = 3.35, *p* = 0.004, *r* = 0.56], and the accuracy of Location-Number Stroop and Updating (color) demonstrated tendencies of improvement [*t*(24) = 2.05, *p* = 0.057, *r* = 0.39; *t*(24) = 1.82, *p* = 0.095, *r* = 0.35]. Training performance of other training tasks did not demonstrate significant increments.

Next, the Group × Time ANOVA analyses revealed the significant Group × Time interaction effect in the Block Design sub-test [*F*(1,49) = 6.69, *p* = 0.013, η_*p*_^2^ = 0.12]. Subsequent paired *t*-tests showed that, although both the training and control groups showed significant improvement in the Block Design test, the training group displayed a greater improvement [training, *t*(24) = 6.84, *p* < 0.001, *r* = 0.81; control, *t*(25) = 2.2, *p* = 0.013, *r* = 0.40]. Paired *t*-tests also demonstrated that the training group had a significantly improved Stroop interference score [*t*(24) = 2.97, *p* = 0.007, *r* = 0.52] and Symbol Span [*t*(24) = 2.426, *p* = 0.023, *r* = 0.44]. By contrast, there was no such change in the control group. Please note that all the *p*-values reported for the improvements in cognitive tests were uncorrected for multiple comparisons, and the significant interaction effect found in the Block Design didn’t survive FDR correction (*p* = 0.091). Results for all neuropsychological tests are presented in Table 5.

**TABLE 5:** Pre- and post-scores for cognitive tests.

	Training group (*n* = 25)				Control group (*n* = 28)				*t* ^†^	*F*	p*
	Pre	Post	*t* ^‡^	p*	Pre	Post	*t* ^‡^	p*			
Stroop	10.18 (7.22)	14.2 (8.26)	**2.97**	**0.007**	9.77 (6.35)	10.93 (10.52)	0.58	0.564	–0.21	1.395	0.243
TMT^††^	1.35 (0.65)	1.48 (0.81)	0.714	0.482	1.62 (0.58)	1.47 (0.84)	–0.8	0.432	1.57	1.144	0.29
SSP	27.92 (4.25)	31.16 (6.16)	**2.43**	**0.023**	28.12 (4.13)	29.15 (4.86)	1.54	0.137	0.17	2.213	0.143
DS	23.48 (3.87)	24.52 (4.03)	1.67	0.108	23.31 (3.06)	23.96 (4.04)	1.1	0.282	–0.18	0.201	0.656
AR	29.28 (1.74)	29.16 (2.49)	–0.32	0.749	28.73 (2.31)	29 (1.88)	0.65	0.521	–0.96	0.488	0.488
BD	55.56 (7.75)	61.48 (4.81)	**6.84**	**<0.001**	55.08 (7.99)	57.42 (6.57)	**2.2**	**0.038**	–0.22	**6.693**	**0.013**
MR	28.84 (2.88)	29.44 (2.99)	0.86	0.4	28.73 (2.86)	29.38 (2.42)	1.07	0.296	–0.13	0.003	0.954

Stroop, Stroop interference score; TMT, trail making test interference score; SSP, symbol span; DS, digit span; AR, arithmetic; BD, block design; MR, matrix reasoning. *Uncorrected for multiple comparisons. ^†^Independent *t*-test to check group differences in baseline performances. ^††^Higher scores indicate worse performance. ^‡^Paired *t*-test to assess the changes between pre- and post-performances. Statistical significances are indicated in bold (*p* < 0.05).

### Graph theoretical analysis

#### Modularity analysis of the brain network organization

**FIGURE 2:**
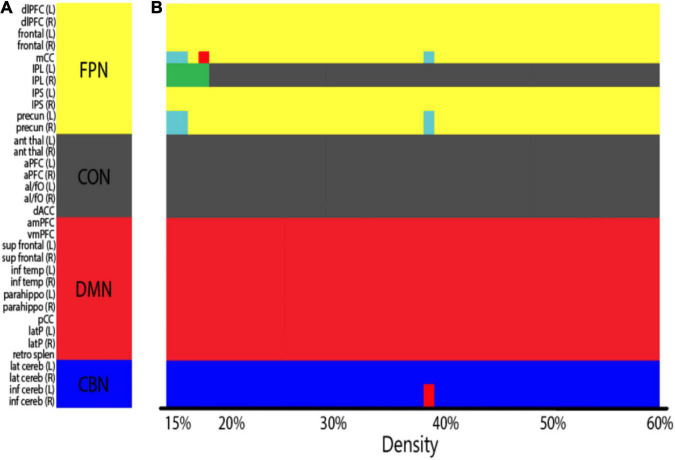
**(A)** Predefined ROI network membership (Table 3; FPN, fronto-parietal network; CON, cingulo-opercular network; DMN, default mode network; CBN, cerebellar network). Nodes are colored by network membership. **(B)** Network organization of all subjects aggregated (both the training and the control groups averaged) as identified by the modularity analysis. *X*-axis indicates network densities. At each density, nodes that were grouped together are coded by the same color. A few nodes were not grouped into one of the four predefined networks at low densities (light blue and green). Overall, subjects showed a similar network organization to the predefined ROI network membership across network densities.

We tested whether cognitive control training promotes the integration of functional networks by calculating the mean degree for each network and examining whether the increases in network degree were greater in the training group than in the control group. Before assigning nodes to each network according to predefined ROI partition schemes (Table 3 and Figure 2A), we first conducted a modularity analysis of all 51 subjects’ averaged connectivity matrices to ensure that the networks of our subjects were organized similarly to the predefined network partitions. The modularity analysis yielded four network partitions across varying network densities (Figure 2B; density ranging 15–60%), which share a structure analogous to that of the predefined network membership (Figure 2A and Table 3). A few nodes were not grouped into one of the four networks at low densities (light blue and green), which may result from low stability and reliability of network measures found at low network densities (Braun et al., 2012; Welton et al., 2015). The overall similarity between the network organization observed in our sample and the predefined network membership allowed us to group nodes according to the predefined network affiliation in subsequent analyses.

#### Change in network functional connectivity after training

**FIGURE 3:**
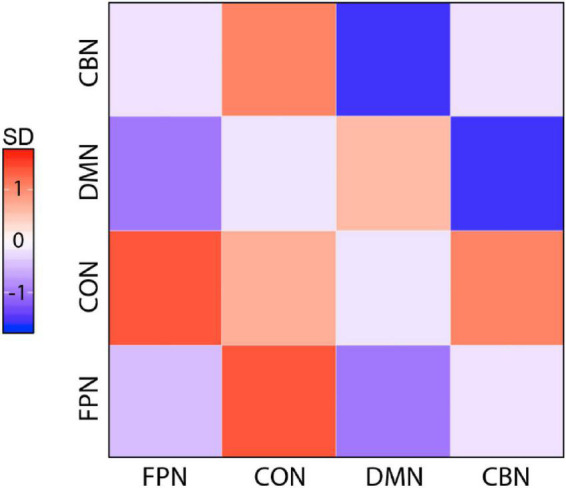
Magnitude of change for all pairwise network FCs in the training group. Magnitude of change is colored by SDs from the mean calculated across all pairs and both groups. Overall, the training group showed a tendency toward increased FC of CON and decreased FC of other three networks compared with the control group (FPN, fronto-parietal network; CON, cingulo-opercular network; DMN, default mode network; CBN, cerebellar network).

First, we performed basic FC analyses to examine the effects of training on network FC. We grouped ROI according to the predefined network membership and tested every pair of networks (4 × 4 pairs) for significant changes in FC after training. Group × Time ANOVAs identified a significant interaction in FC change between DMN and CBN, but this interaction occurred due to significant baseline differences in DMN–CBN FC between the two groups (*p* = 0.049). The remaining pair of networks by paired *t*-tests of the training group showed no significant changes in FC between these two networks (*p* = 0.138) or in all other pairs of networks. Although the visual inspection of matrix plots for change in network FC in the training group suggested that the training group generally showed a tendency toward increased FC of the CON and decreased FC of other three networks compared to the control group (Figure 3), a training effect was not observed in FC analyses of individual pairs of networks.

#### Change in network degree after training

**FIGURE 4:**
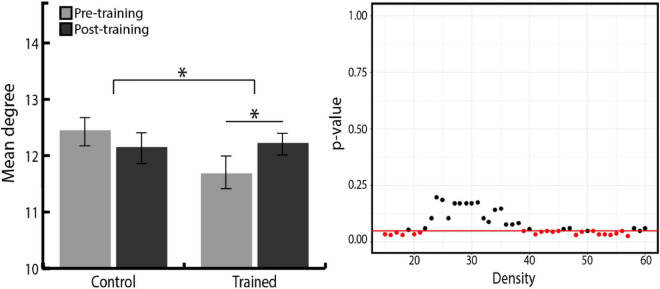
**(Left)** Density-averaged CON degree in both groups, before and after training. Density-averaged CON degree significantly increased in the training group compared with the control group (*p < 0.05). Paired *t*-tests showed that the CON degree significantly increased in the training group. There was no significant difference between the two groups in initial CON degree (*p* = 0.054). Error bars indicate the SEM. **(Right)** *P*-value distribution for Group × Time ANOVA of the CON degree across network densities. The red line indicates a *p*-value of 0.05, and red dots indicate the densities at which the difference in the degree change between two groups is significant.

We further investigated the degree measure to find networks that increased their number of links across all networks after training. Group × Time ANOVA revealed that among four networks, the degree of the CON significantly increased in the training group compared with the control group (Figure 4, right). However, as shown in Figure 4, the statistical significance of this training effect fluctuated according to varying network thresholds. To overcome the arbitrary bias in thresholding and acquire representative statistics, we averaged the degree of each network across the range of network densities and used these mean values in subsequent analyses. Even with this method, the degree of all nodes averaged across densities was positively correlated with the degree of all nodes at each network density, suggesting that our results were robust to any biases in thresholding (Marek et al., 2015). Group × Time ANOVA was performed again with the density-averaged degree of each network, which confirmed the significant Group × Time interaction effect [*F*(1,49) = 4.09, *p* = 0.049, η_*p*_^2^ = 0.08; Figure 4, left]. Follow-up paired *t*-tests showed that the CON degree significantly increased in the training group [*t*(24) = –2.01, *p* = 0.028, *r* = 0.38, one-tailed], whereas no change was observed in the control group. Density-averaged degree in other networks showed no significant Group × Time interaction in Group × Time ANOVA (FPN, *p* = 0.206; DMN, *p* = 0.817; CBN, *p* = 0.539).

**FIGURE 5:**
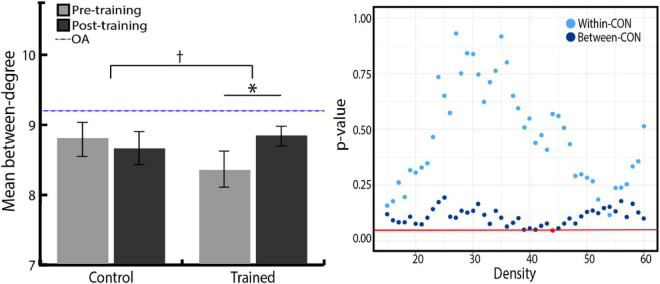
**(Left)** Density-averaged between-CON degree in both groups, before and after training. Between-CON degree significantly increased in the training group (*p < 0.05), although the increase was not significantly greater compared with the control group (^†^ *p* < 0.10). There was no significant difference between the two groups in initial between-CON degree (*p* = 0.201). The blue dashed line represents between-CON degree of older adolescents from ADHD-200 dataset. Error bars indicate the SEM. **(Right)** *P*-value distribution for Group × Time ANOVA of the CON within and between degree across network densities. The red line indicates a *p*-value of 0.05, and red dots indicate the densities at which the difference in the degree change between two groups is significant.

Since we found that the CON degree significantly increased after training, we further investigated whether it was due to the increase in the number of links within CON, or links between CON and other networks. To this end, Group × Time ANOVA was conducted separately for the change in within-CON degree (the number of links within nodes of CON) and the change in between-CON degree (the number of links between nodes of CON and those of the other three networks). We found a trend toward an increase in between-CON degree in the training group compared with the control group, whereas there was no difference between the two groups in the within-CON degree (Figure 5, right). Again, to remove the bias in network thresholding, we performed Group × Time ANOVA with the density-averaged degree. We found the tendency toward Group × Time interaction, but the result was not statistically significant [*F*(1,49) = 3.23, *p* = 0.078, η_*p*_^2^ = 0.06; Figure 5, left]. However, follow-up paired *t*-tests showed that the between-CON degree significantly increased in the training group [*t*(24) = –2.01, *p* = 0.027, *r* = 0.38, one-tailed], whereas no change was observed in the control group. Regarding within-CON degree, neither the training group (pre = 3.33 ± 0.61, post = 3.38 ± 0.84, *p* = 0.79) nor the control group (pre = 3.64 ± 0.76, post = 3.48 ± 0.68, *p* = 0.37) showed significant change in density-averaged values. Therefore, the increased degree of CON in the training group seems to be driven more by an increase in between-network links than within-network links.

We then investigated which networks had increased links to the CON after training. We calculated the average number of links between the CON and other three networks, and examined whether there was any particular network showing increased number of links with the CON after the training. We found that no individual network had a significantly increased number of links with the CON in the training group paired *t*-test (FPN, *p* = 0.447; DMN, *p* = 0.222; CBN, *p* = 0.824). Therefore, the increase in between-CON degree in the training group was not attributed to increased links with any particular network but to an increase in its overall connectivity with other networks.

In addition, in order to examine the contribution of a particular region to network integration, which is ignored when averaging at the network level, we tested all 34 ROI for significant changes in between-network links after training. We investigated which brain regions had increased or decreased numbers of links to regions belonging to networks other than their own. Among the 34 ROI, only one ROI (the right inferior cerebellum) was found to have significant changes in both the Group × Time ANOVA [i.e., significant interaction; *F*(1,49) = 5.14, *p* = 0.028, η_*p*_^2^ = 0.10] and the follow-up training group paired *t*-test [*t*(24) = 2.26, *p* = 0.033, *r* = 0.41]; this cerebellar region showed greater decreases in its between-network links with other networks in the training group compared with the control group. Two groups did not show any difference in changes in the number of within-network links of this region (*p* = 0.59). Notably, no ROI belonging to CON showed training-related changes in between-network links. This result again indicates that the increase in between-CON degree was not driven by increased links of a particular CON region but by an increase in the overall CON connectivity.

#### Difference in network degree between young and older adolescents

Since we observed training-related increases in between-CON degree, we sought to evaluate the meaning of this observation in light of the developmental changes occurring in adolescent brain networks. To this end, we examined whether there was any difference in network degree between older adolescents from the ADHD-200 dataset and our younger adolescents before training. We performed independent *t*-tests to compare the total, within, and between degree of each network between young and older adolescents. Compared with our young adolescents, older adolescents showed smaller within-CON degree [*t*(107) = 3.90, *p* ≤ 0.001, *r* = 0.35] and greater between-CON degree [*t*(107) = –2.59, *p* = 0.011, *r* = 0.24; Figure 5, right, blue dashed line]. There was no difference in the degree of the other networks between young and older adolescents, except for a trend of smaller total-CBN and between-CBN degree in the older adolescents compared to the young adolescents (Table 6).

**TABLE 6:** Difference in network degree between young and older adolescents.

	Total degree			Within degree			Between degree		
	YA	OA	*p*	YA	OA	*p*	YA	OA	*p*
FPN	13.52 (1.23)	13.63 (1.13)	0.629	5.60 (0.93)	5.53 (0.93)	0.715	7.92 (0.71)	8.10 (0.70)	0.198
CON	12.08 (1.44)	12.13 (1.15)	0.841	3.49 (0.7)	2.99 (0.64)	**<0.001**	8.59 (1.27)	9.14 (0.95)	**0.011**
DMN	11.83 (1.23)	11.94 (1.25)	0.622	4.53 (0.95)	4.66 (1.05)	0.487	7.30 (0.66)	7.28 (0.69)	0.899
CBN	11.38 (1.83)	10.64 (2.24)	0.063	1.97 (0.65)	1.91 (0.72)	0.652	9.42 (1.76)	8.74 (1.87)	0.053

YA, young adolescents; OA, older adolescents; FPN, fronto-parietal network; CON, cingulo-opercular network; DMN, default mode network; CBN, cerebellar network. Statistical significances are indicated in bold (*p* < 0.05).

Having observed the difference between younger and older adolescents in between-CON degree where the training effect was also revealed, we further examined whether the training group caught up to older adolescents in between-CON degree after training. Independent *t*-tests found the significant difference in between-CON degree between the training group and the older adolescents at pre-training [*t*(81) = 3.12, *p* = 0.002, *r* = 0.33], but the difference disappeared at post-training [*t*(81) = 1.37, *p* = 0.174, *r* = 0.15; depicted in the Figure 5 on the left side as reduced gaps between pre- and post-training bars of the training group and the blue dashed line].

#### Correlations between changes in network degree and changes in performance on cognitive tests

**FIGURE 6:**
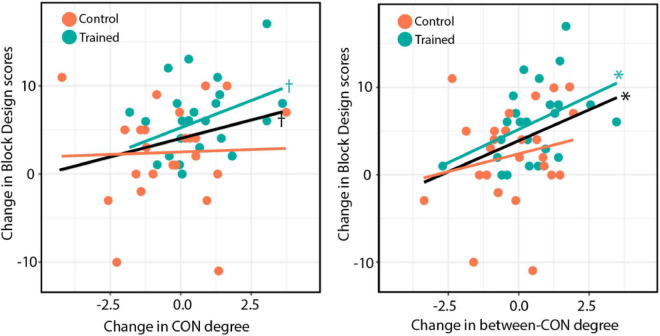
**(Left)** Changes in total-CON degree for individual subjects across both groups plotted against changes in Block Design test performance after training. **(Right)** Changes in between-CON degree plotted against changes in Block Design test performance after training. The black line indicates the regression line for both groups, whereas the blue and orange lines indicate the regression lines for the training and control groups, respectively. The results of significance tests are indicated beside the corresponding regression lines (*p < 0.05; ^†^*p* < 0.10).

Finally, we tested whether the observed changes in the total-CON degree and the between-CON degree were associated with improvements in cognitive test performance. We found that the change in between-CON degree was positively correlated with the improvement in the Block Design test in both groups combined [*r* = 0.36, *t*(49) = 2.67, *p* = 0.01; Figure 6, right, black line] as well as in the training group alone [*r* = 0.43, *t*(23) = 2.29, *p* = 0.03; Figure 6, right, blue line], but not in the control group [*r* = 0.21, *p* = 0.31; Figure 6, right, orange line]. No slope difference was found between the two groups (*z* = 0.84, *p* = 0.40). The change in total-CON degree also showed a trend of positive correlation with the improvement in Block Design test in both groups combined [*r* = 0.25, *t*(49) = 1.80, *p* = 0.078; Figure 6, left, black line] as well as in the training group alone [*r* = 0.38, *t*(23) = 1.98, *p* = 0.06; Figure 6, left, blue line], but not in the control group (*r* = 0.04, *p* = 0.86; Figure 6, left, orange line). There was no slope difference between the two groups (*z* = 1.22, *p* = 0.22).

**FIGURE 7:**
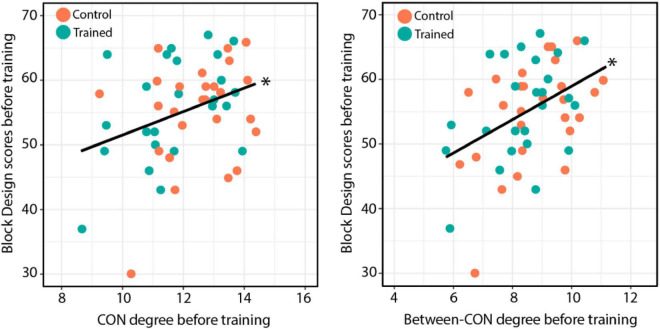
**(Left)** Total-CON degree for individual subjects across both groups plotted against Block Design test performance before training. **(Right)** Between-CON degree plotted against Block Design test performance before training. The results of significance tests are indicated beside the corresponding regression lines (*p < 0.05).

We also tested whether the total-CON degree and between-CON degree were associated with performance in the Block Design test *before* training. We found that both the total-CON degree [*r* = 0.332, *t*(49) = 2.463, *p* = 0.017; Figure 7, left] and between-CON degree [*r* = 0.42, *t*(49) = 3.25, *p* = 0.002; Figure 7, right] were positively correlated with Block Design test scores before training. Other cognitive tests showed no such correlations with total-CON degree and between-CON degree (data not shown).

The right inferior cerebellum, the individual ROI that was found to have significant changes in the training group revealed the correlation between its decreased between-network links and the improvements in Block Design scores [*r* = –0.29, *t*(49) = –2.10, *p* = 0.04].

#### Change in network participation coefficient after training

As we identified training-related increases in between-CON degree, we investigated whether a comparable result was observed with a related graph measure of network integration, participation coefficient (PC). We calculated the mean PC for each of the four networks and examined whether the two groups showed any difference in the network PC change after training. In contrast to the increases in between-network degree, the increases in network PC were not significantly greater in the training group compared with the control group (Supplementary Analysis and Supplementary Figure 1).

## Discussion

In this study, we found that the integration of functional networks was enhanced as a result of multicomponent cognitive control training as captured by increased total- and between- cingulate-opercular network (CON) degree. The increase in between-CON degree was not attributable to increased connectivity of a particular network or a particular CON region. The analysis of the network degree of older adolescents revealed that older adolescents had a smaller within-CON degree and a greater between-CON degree than young adolescents. Regarding the brain–behavior relationship, increases in between-CON degree after training were associated with improvements in the Block Design test performance.

### Increased cingulo-opercular network degree after training and with age

Adolescence is a unique period, during which the brain undergoes dynamic changes at many different levels to support the transition to adult-level cognition. At the systems level, brain networks become more involved in the integration of information from widely distributed regions, yielding more complex cognitive abilities such as cognitive control (Stevens, 2016; Grayson and Fair, 2017). Although these changes are thought to be driven by the interplay between developmental programs and environmental inputs (Greenough et al., 1987), the way in which these two factors interact with one another is poorly understood.

In this study, we tested the hypothesis that cognitive control training would accelerate typical network development during adolescence and lead to more integrated networks. To this end, we applied graph theoretical analysis to resting-state FC data obtained from young adolescents who undertook 6 weeks of cognitive control training, and to an independent dataset from typically developing older adolescents. The results were consistent with previous characterizations of major changes in functional networks occurring throughout development. Studies have shown that fundamental properties of functional networks, such as network organization, are established early in development, whereas the capacity for networks to integrate develops into adulthood (Shaw et al., 2008; Menon, 2013). It is noteworthy that the smaller within- and greater between-links of CON observed in older adolescents are consistent with the findings of a previous report demonstrating that there is a continuous integration of CON with other networks during adolescence (Marek et al., 2015). This increased integration of CON has been shown to play a critical role in age-related improvements in cognitive control. Functionally, CON is involved in set-maintenance, sustained alertness, and adjustments for feedback during task performance (Dosenbach et al., 2008; Coste and Kleinschmidt, 2016). Given that cognitive control is underlain by the interaction among separable cognitive components, the integration of CON with other networks may facilitate stable communication among these components, boosting behavioral performance in cognitive control tasks (Velanova et al., 2008). Our results suggest that this process of integration could be further advanced by extra training. Also, the observation that cognitive training only had an impact on CON, but not on other networks, suggests that the enhanced integration could be driven by interactions between developmental processes and relevant learning. It is well known that greater neuronal plasticity in children and adolescents allows their neuronal networks to reorganize more readily in response to environmental stimulation and to better adopt new skills and information (Johnston, 2009). The effects of training appear to benefit from this greater neuronal plasticity (Qin et al., 2004), supposedly making CON more susceptible to learning-related changes during adolescence.

Challenging the traditional view of development and learning as separate domains, researchers increasingly believe that those are interactive and complementary processes (Karmiloff-Smith, 1994; Stiles, 2008). For instance, it has been suggested that development and learning are not separable constructs, but are two ends of the same continuum, both process shaped by experience-expectant and experience-dependent mechanisms (Galván, 2010). In this view, development is not a passive process dictated by a genetic instruction, but is considered as self-organizing and activity-dependent processes involving an interplay with the environment (Johnson, 2011). Indeed, learning and experience shape, mold, and sculpt developmental changes through activity-dependent creation and refinement of synapses (Stellwagen and Shatz, 2002). In this regard, it is possible that certain cognitive processes can be developmentally sped up with training or delayed with adverse experience (Needham et al., 2002; James, 2010). Then how does intrinsic FC reveal neural mechanisms that support these accelerated developmental experiences? When we engage in certain behaviors, evoked neural activity causes perturbations to intrinsic connectivity by coupling different neural components, perturbations that persist across multiple timescales (Han et al., 2008; Harmelech et al., 2013). Thus, an individual’s intrinsic network configuration at one time point reflects a history of past behavior and environmental inputs (Tambini et al., 2010; Sadaghiani and Kleinschmidt, 2013). From this perspective, developmental changes in brain networks can be understood as emerging from a child’s experience that involves active sampling of the external world over the course of development (Berkes et al., 2011; Byrge et al., 2014). Then cognitive training, by providing environmental inputs that are extra and additional to those generally available for children, provides trained children with age-linked experiences more precociously and intensively. This line of reasoning could explain why training-induced and growth-associated changes in intrinsic networks might possibly converge to a large degree, generating an additive effect.

### The differential impact of training on between- versus within- cingulo-opercular network degree

Questions remain, however, as to why the training influenced one aspect of the developmental change, namely the increase in between-CON degree, but did not have an impact on the decrease in within-CON degree in this study. A plausible explanation may come from the nature of cognitive control, the particular cognitive domain upon which the current training was designed to focus. As described above, cognitive control relies on the coordination of several cognitive components, which is supported by the cooperation between diverse brain networks. Thus, the ability for the brain to transfer and integrate information across networks may be more pivotal than local communication within networks (Cocchi et al., 2013). Indeed, it has been shown that within-network communication is important for simple cognitive tasks such as motor execution, whereas between-network communication is critical for more complex cognitive skills such as working memory, which requires the engagement of multiple functions (Cohen and D’Esposito, 2016). Researchers have proposed that CON serves as a mediator for competitive interactions between FPN and DMN, playing a crucial role in facilitating crosstalk between networks (Sridharan et al., 2008; Bressler and Menon, 2010). Taken together, repeated exposures to cognitive control tasks through training tended to recruit the between-network interactions involving CON rather than interactions within CON, which appeared to remain relatively separated from the demands of the training. In line with these observations, many training studies have found that the most relevant changes in resting-state FC occur between, rather than within, networks after training (Lewis et al., 2009; Astle et al., 2015; Dresler et al., 2017). Our results suggest that these training-induced interactions between CON and other networks may coincide with the developmental change, leaving a stronger trace in the increasing between-CON links than in the decreasing within-CON links.

### Change in network participation coefficient after training

The weak effects observed in changes in PC with training in this study may be explained by the differential impact of training on between-CON versus within-CON links. Note that PC is a function of the number of within-network links as well as the number of between-network links (Supplementary Analysis). In general, the increase in PC of nodes occurs when links that were highly concentrated in a single network are redistributed to other less-connected networks, yielding a more even distribution of links across networks. A robust increase in PC is most often observed when the increase in nodes’ between-network links are accompanied by a corresponding decrease in their within-network links. This does not seem to be the case for the training effects found in this study, given that we observed an increase in between-CON degree following training, but the within-CON degree was unaffected. This stable within-CON degree suggests that the training led to increases in PC in the training group compared with the control group that were insufficient to reach significance level in the group and time interaction, even though a slight trend toward increased PC was observed in the training group. The differential effects of training on between-CON versus within-CON degree are possibly due to the cognitive demands imposed by the cognitive control tasks, and thus could reflect a characteristic of our training.

### Correlation between cingulo-opercular network degree and Block Design score

Importantly, we found an association between brain changes and behavioral changes following the training. This suggests that the observed change in between-CON degree was induced by training, not by some unknown factors, with hints of individual differences in neural changes that reflect individual differences in behavioral gains. Surprisingly, CON degree was associated with performance in Block Design tests before training, but not with other behavioral tests. This result may account for the weaker training effects observed with the other behavioral tests. It has long been known that the improvement of certain cognitive skills could be constrained by the level of brain maturation (Jolles and Crone, 2012). For example, the speed of information processing is modulated by the maturation of white matter (Nagy et al., 2004; Fields, 2008), which could in turn, for instance, constrain practice-related gains on higher level cognition such as working memory (Fry and Hale, 1996). In a similar vein, subjects in our study are at a developmental point when CON becomes more integrated with other networks. Thus, our findings that this developmental process could be modified by training may set limits on which cognitive performance is prone to be affected by training. In other words, the interaction between brain development and training may alter the more developmentally responsive networks, in this case CON, and the change in CON could in turn affect associated cognitive performance, in this case Block Design test performance. The fact that training-related behavioral gains could be limited by the level of brain maturation may shed some light on the debate of the effectiveness of cognitive training (Melby-Lervåg and Hulme, 2013).

### Decreased between-network links of the right inferior cerebellum after training

Notably, the right inferior cerebellum had a decreased number of links with other networks after training. We found that the decrease in between-network links in this region mirrored the improvements in Block Design scores in both groups combined. This finding is consistent with the hypothesis that a critical role of the cerebellum is adaptive control and experience-driven plasticity (Caligiore et al., 2017). Previous studies have demonstrated that the cerebellum contributes to experience-driven learning by modulating cortical activities through synaptic connections within a cerebro-cerebellar loop (Albert et al., 2009; Vahdat et al., 2011; Sami et al., 2014). Although few studies have examined learning-dependent changes in cerebro-cerebellar communication in a cognitive control domain, one human imaging study has reported decreased cerebro-cerebellar interaction after response inhibition learning, as revealed by psycho-physical interaction analysis (Hirose et al., 2014). Our finding of a decreased number of links between a cerebellar region and other cortical networks similarly demonstrates the function of modified cerebellar-cortical connections in guiding the experience-driven learning of control functions. In addition, researchers have found that the cerebellum exhibits resting-state networks mapped onto those found in the cortex (O’Reilly et al., 2010; Buckner et al., 2011). These functional maps of the cerebellum are dominated by association networks involved in adaptive control, and show both common and variable organization across individuals, thought to reflect their roles in individual-specific plasticity in cognitive control (Marek et al., 2018). We found that the right inferior cerebellum used in this study (*x, y, z* = 20, –87, –33) belongs to the DMN of cerebellar networks (Buckner et al., 2011). Although it is unclear how cerebellar DMN coordinates with cortical networks to support adaptive control, the altered connectivity between the cortical DMN and other cortical control networks has been well documented during control-related learning, usually in the form of increased dissociations (Takeuchi et al., 2013, 2014). It is an interesting question whether our finding of the decreased links between cerebellar DMN and other cortical networks can be interpreted in light of this previously reported relationship between the cortical DMN and other networks. It is also noteworthy that in our study older adolescents showed a trend of smaller between-CBN degree compared with young adolescents (Table 5). This result is consistent with our main findings of the potential synergistic interaction between development and training. Nevertheless, in order to clarify these issues, future studies are needed to elucidate the role of specific cerebellar networks in cognitive processes and how they contribute to cognitive maturation in an experience-dependent manner.

### Implications and limitations

Adolescence is characterized by certain psychological traits such as increases in sensation and novelty seeking that are linked to specific brain maturational changes (Steinberg, 2004; Casey et al., 2008). Also, cognitive control is impaired in many psychiatric disorders, which is also apparent in the adolescent period albeit in attenuated form (Paus et al., 2008). Thus, this line of research may help target interventions to ameliorate adolescent maladaptive behaviors and prevent or remediate the emergence of psychopathology.

Several caveats, however, are worth mentioning. Although it may be difficult to find strong effects from short-term behavioral intervention that was implemented for a relatively short period of time, especially on highly plastic adolescent brains going through substantial maturational and developmental changes, the observed changes were modest and in some cases did not reach statistical significance. Some of the results reported in the current study as indications of training effects fell short of the statistical significance level of *p* = 0.05; the change in between-CON degree showed a trend toward interaction (*p* = 0.078) and the correlation between changes in CON degree and changes in Block Design score didn’t reach the significance (*p* = 0.078 in both groups combined and *p* = 0.06 in the training group alone). Also, the statistical significance of the improvement in the Block Design test after training suffers when subject to the correction for multiple comparisons (FDR-corrected *p* = 0.091). The overall effects found in the current study are only moderate (Karbach and Verhaeghen, 2014), which requires the evidence presented in the current study and its interpretations to be accepted with caution.

Second, using non-binary, weighted measures of brain connectivity failed to demonstrate the significant training effects in the study, as described in the analyses of FC (see section “Change in network functional connectivity after training”) and participation coefficient (see section “Change in network participation coefficient after training”). Although the results from these analyses yielded patterns matched by what was found with the binary, unweighted measure of connectivity (i.e., degree), it needs to be stressed that the analysis of degree comes with the loss of weight information to a certain degree, which may contribute to the discrepancy in findings from FC and participation coefficient analysis.

Third, the resting-state scans were preceded by the task fMRI scans in the study. Indeed, some studies have shown that a task before an rs-fMRI scan can alter the resting-state FC over the short term (Waites et al., 2005; Stevens et al., 2010). However, since the order of scans was the same for both groups, it is unlikely that the task influenced the resting-state FC of the training group disproportionately. Fourth, it has been pointed out that degree is not an accurate measure of network integration, since degree-based measures of integration are confounded by the size of networks. That is, degree-based approaches are biased toward identifying the nodes of larger networks as hubs for network integration (Power et al., 2013). However, this weakness does not affect our study, since degree-based measures of integration are problematic only when a researcher aims to determine the importance of individual nodes (networks) relative to other nodes (networks) in facilitating network integration. Our study, however, has a repeated measures design; our goal was to track changes in network degree before and after training, no matter which network plays the central role in network integration at any time point. Post-training, the CON was found to be more integrated with other networks than pre-training as demonstrated by increased numbers of links to other networks. The previously reported limitations of degree-based measures, although worth considering, therefore do not apply to our analyses of training effects.

Finally, Korean children already experience much practice with cognitive control in school and everyday lives, and this might have masked the training effect. This explanation relates to the time displacement hypothesis, which states that training should be evaluated in relation to the activities it displaces (Bavelier et al., 2010). However, there is no reason to assume that the training group received greater environmental inputs tapping into cognitive control except for the required training. Also, the task difficulty of training was adapted to tax a great deal of cognitive resources in adolescents, creating significant differences in cognitive demands between the two groups.

One interesting future direction is to unravel the structural basis of the documented changes in functional networks. The changing dynamics of functional networks during adolescence is accompanied by a series of structural alterations including gray matter thinning (Gogtay et al., 2004), synaptic pruning (Petanjek et al., 2011), and an increase in white matter integrity (Lebel et al., 2008). In particular, the increased white matter tract integrity allows faster neuronal transmission, which is thought to underlie the integration of functional networks (Simmonds et al., 2014). Thus, the increased CON links observed in the current study are expected to be paralleled by concomitant changes in white matter networks. Recent studies have demonstrated alterations in structural networks as a result of cognitive training (Caeyenberghs et al., 2016; Román et al., 2017) which are consistent with the function-structure correspondence. In the future, we will explore this possibility by integrating the functional investigation with analyses of structural data, and examine how changes in intrinsic connectivity relate to changes in white matter networks.

## Conclusion

This study, to our knowledge, is the first to explore the effects of cognitive training regarding intrinsic brain connectivity in typically developing adolescents, which found that cognitive control training enhanced the integration of functional networks and facilitated network development. Most training studies with developing children or adolescents to date have not evaluated the training effects in light of the participants’ developmental trajectory. This is crucial given that childhood and adolescence are characterized by great and systematic brain malleability. Network perspective plays a critical role in elucidating such plasticity, because developmental and learning-induced changes emerge in a dialog between brain regions (Bressler and Menon, 2010; Johnson, 2011). Our finding that the training-related integration of brain network is not captured by changes in a single region stresses the importance of analyzing information conveyed at the network level.

## Data availability statement

The raw data supporting the conclusions of this article will be made available by the authors, without undue reservation.

## Ethics statement

The studies involving human participants were reviewed and approved by Seoul National University. Written informed consent to participate in this study was provided by the participants or their legal guardian/next of kin.

## Author contributions

DL and JC contributed to the conception and the design of the study. DL organized the database. RL and SK performed the statistical analysis. RL wrote the first draft of the manuscript, while JC wrote the final revision. RL, SK, and DL wrote sections of the manuscript. All authors contributed to the manuscript revision and read and approved the submitted version.

## Footnotes

1
www.fil.ion.ucl.ac.uk/spm/software/spm12/

2
http://www.nitrc.org/projects/artifact_detect/
