# Supplementary material for: Cognitive control training enhances the integration of intrinsic functional networks in adolescents
Source: Front Hum Neurosci. 2022 Nov 24;16:859358. doi: 10.3389/fnhum.2022.859358 (PMC9729882; doi:10.3389/fnhum.2022.859358)
Supplement: Supplementary file 1 [file Data_Sheet_1.pdf]

Supplementary analysis

Changes in network participation coefficient after training

In addition to network degree, we used another graph theoretical construct, participation coefficient (PC), to examine the effects of training on network integration. PC formalizes and extends the distinction between within-network degree and between-network degree. It assesses the extent to which a node has between-network links compared with within-network links: $y_i = 1 - \sum_{n \in N} \left(\frac{k_i(n)}{k_i}\right)^2$, where N is the set of networks and $k_i(n)$ is the number of links between i and all nodes in the network n (Guimerà and Nunes Amaral, 2005). The PC of a node increases from 0 to 1 as the number of links to other networks increases in comparison with links to nodes within the network. Therefore, a network containing nodes with high PC values is likely to promote the integration of networks.

To investigate changes in network PC after training, we calculated the mean PC for the FP, CO, CB, and DM networks for each subject. We took the absolute value of all negative weights and thresholded each subject's connectivity matrices by network density, ranging from the strongest 15%–60% of pairwise connections. Connections above the threshold were binarized and the PC for every node was calculated and averaged within each network. Group × Time ANOVA was then conducted to examine whether the increases in the mean PC of networks were greater in the training group than in the control group.

We found no significant Group × Time interactions for PC in any of the four networks, including CON (Fig. S1, left). The density-averaged PC of CON confirmed that there were no substantial differences in training-related changes between the two groups (Fig. S1, right), although paired *t*-tests showed that a trend toward increased PC was observed in the training group ($t(24) = -1.48$, $p = 0.076$, $r = 0.29$, one-tailed) while negligible changes were found in the control group ($t = 0.53$, $p = 0.602$). Therefore, the training effects were only apparent as increases in between-network degree, with the effects on increases in PC being limited. The interpretation of these results is addressed in the Discussion.

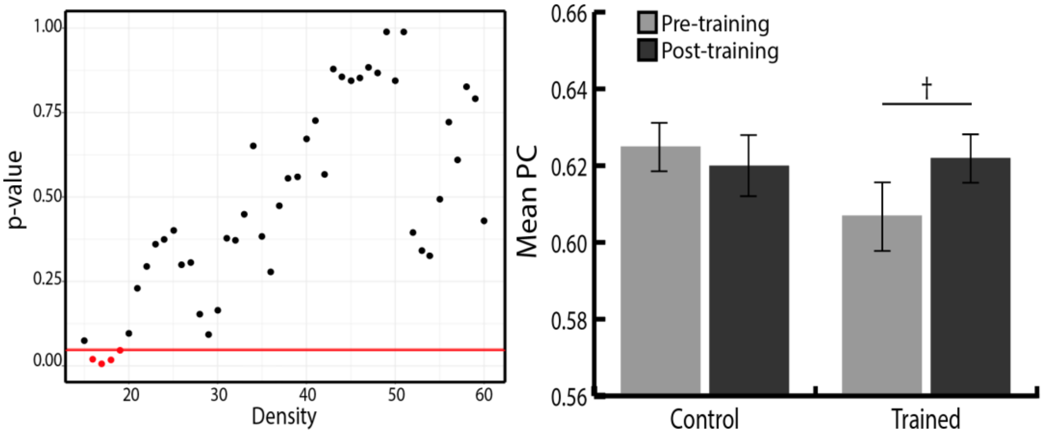

Figure S1. Left: *P*-value distribution for Group × Time ANOVA of the CON PC across network densities. The red line indicates a *p*-value of 0.05, and red dots indicate the densities at which the difference in the degree change between two groups is significant. **Right**: Density-averaged CON PC in both groups, before and after training. There was no significant difference in changes in CON PC between the two groups, although a slight trend toward increased PC was observed in the training group († $p = 0.076$). Initial CON PC was not different between the two groups ($p = 0.094$). Error bars indicate the SEM.

References

Guimerà, R., & Nunes Amaral, L.A. (2005). Functional cartography of complex metabolic networks. *Nature*, *433*, 895–900.
